# Intermediate conductance Ca^2+^-activated potassium channels are activated by functional coupling with stretch-activated nonselective cation channels in cricket myocytes

**DOI:** 10.3389/finsc.2022.1100671

**Published:** 2023-01-23

**Authors:** Tomohiro Numata, Kaori Sato-Numata, Masami Yoshino

**Affiliations:** ^1^ Department of Integrative Physiology, Graduate School of Medicine, Akita University, Akita, Japan; ^2^ Department of Biology, Tokyo Gakugei University, Tokyo, Japan

**Keywords:** IK channel, mechano-sensitive channel, patch-clamp technique, cricket (*Gryllus bimaculatus*), oviduct, myocyte, functional coupling

## Abstract

Cooperative gating of localized ion channels ranges from fine-tuning excitation–contraction coupling in muscle cells to controlling pace-making activity in the heart. Membrane deformation resulting from muscle contraction activates stretch-activated (SA) cation channels. The subsequent Ca^2+^ influx activates spatially localized Ca^2+^-sensitive K^+^ channels to fine-tune spontaneous muscle contraction. To characterize endogenously expressed intermediate conductance Ca^2+^-activated potassium (IK) channels and assess the functional relevance of the extracellular Ca^2+^ source leading to IK channel activity, we performed patch-clamp techniques on cricket oviduct myocytes and recorded single-channel data. In this study, we first investigated the identification of IK channels that could be distinguished from endogenously expressed large-conductance Ca^2+^-activated potassium (BK) channels by adding extracellular Ba^2+^. The single-channel conductance of the IK channel was 62 pS, and its activity increased with increasing intracellular Ca^2+^ concentration but was not voltage-dependent. These results indicated that IK channels are endogenously expressed in cricket oviduct myocytes. Second, the Ca^2+^ influx pathway that activates the IK channel was investigated. The absence of extracellular Ca^2+^ or the presence of Gd^3+^ abolished the activity of IK channels. Finally, we investigated the proximity between SA and IK channels. The removal of extracellular Ca^2+^, administration of Ca^2+^ to the microscopic region in a pipette, and application of membrane stretching stimulation increased SA channel activity, followed by IK channel activity. Membrane stretch-induced SA and IK channel activity were positively correlated. However, the emergence of IK channel activity and its increase in response to membrane mechanical stretch was not observed without Ca^2+^ in the pipette. These results strongly suggest that IK channels are endogenously expressed in cricket oviduct myocytes and that IK channel activity is regulated by neighboring SA channel activity. In conclusion, functional coupling between SA and IK channels may underlie the molecular basis of spontaneous rhythmic contractions.

## 1 Introduction

Mammals and invertebrates use the combinatorial function of numerous ion channels expressed in muscle cells to control the electrical properties of membranes. Elucidating their respective roles is essential to understanding the rhythmic contraction mechanisms produced by muscle tissue.

As a model system for the investigation of these problems, we used striated muscle cells from the oviduct of crickets, building on previous pioneering work in insects, such as fruit flies and locusts (1–16).

One of the critical functions of oviposition is the repeated contraction and relaxation of the oviduct visceral muscles during egg transport. The rhythmic contraction in oviductal muscle cells allows the egg to pass through the oviduct (4, 7, 9, 17). Similar rhythmic contractions occur in tubular tissues, including the gastrointestinal and urogenital tracts of most mammals (18–21). Although these contractile processes require the analysis of simple systems consisting of mechanically stimulated muscle contractions (7, 10), the molecular physiological mechanisms still need to be fully understood.

A defined group of ion channels, including intermediate conductance Ca^2+^-activated potassium (IK) channels, KCa3.1 (also known as KCNN4, IKCa, SK4) belongs to the Ca^2+^-activated K^+^ channel (K_Ca_) family among Ca^2+^-sensing proteins (22–24), are functionally linked in mediating ion fluxes that influence the membrane potential that produces periodicity. Moreover, it has long been recognized that these membrane potential changes affect ion channel gating (25–27). In vertebrates, IK channels are highly expressed in epithelial cells, the central nervous system, and in tumor-type tissues, such as that of cervical cancer (28, 29). In invertebrates, functional expression of the IK channel is only observed in the cranial nerves of the cockroach *Periplaneta americana* (30), the muscle tissue of the locust *Schistocerca gregaria (* 31), and the body-wall muscle of the *Drosophila melanogaster* (32).

*Drosophila melanogaster* is an important model organism for genetics and molecular biology and has accumulated genetic information, including K_Ca_ channels, for more than 100 years. The K_Ca_ channel gene family consists of two groups, the BK (*dSlo*, *SLO2*) group and the SK group (*dSK*), which includes IK (33, 34).

Mutant phenotypes of BK channels are often associated with negative regulation of ganglionic synapses, and *dSlo* mutants play a role in neuronal function, abnormal circadian activity, and locomotor disorders (35–37). In flight muscles, the mutant disrupts the homeostatic adjustments in neural circuits at the flight initiation (38, 39).

Mutant phenotypes of SK channels are involved in neuronal activities such as resting membrane potential, synaptic transmission, and synaptic plasticity, resulting in learning and memory, visual reception, sensory and motor deficits (40–46). Knock down of SK in class IV sensory neuron induces faster heat avoidance behavior probably due to an alteration of firing properties *via* destruction of coordination between L-type voltage-gated Ca^2+^ channels and SK channels (43, 44).

The molecularphysiological importance of IK channels in oviduct muscle provides new insights into muscle contraction mechanisms in addition to flight and body wall muscles.

Recently, we have shown that mechanical stretching-dependent Ca^2+^ influx from the plasma membrane occurs in the development of spontaneous rhythmic contractions in the cricket oviduct (9). Furthermore, we showed that this contraction is closely associated with rhythmic membrane hyperpolarization (RMH) (47). RMH involves a Gd^3+^-sensitive extracellular Ca^2+^ influx pathway, which is consistent with the properties of stretch-activated (SA) ion channels mentioned in our previous report (15).

In this study, we characterized endogenously expressed IK channels in the cricket oviduct. Second, we investigated Ca^2+^-mediated functional coupling of SA and IK channels involved in forming rhythmic hyperpolarization.

## 2 Materials and methods

### 2.1 Insect rearing

*Gryllus bimaculatus* used in the experiments were sexually mature females purchased from a local pet store as food for pet reptiles, and thus, genetic and environmental variability in the specimens were limited. The crickets were housed in plastic containers with cardboard shelters until further analysis. All crickets were bred at 27 ± 2°C, with humidity of 35–60%, in a 12:12-h light:dark cycle. Crickets were provided *ad libitum* access to feed and water for insects (I, Oriental yeast CO., LTD., Kyoto, Japan).

### 2.2 Cell isolation

The adult female crickets were fixed in a chamber *via* the upper dorsal area under CO_2_ anesthesia. The lateral oviducts were exposed by removing connective tissue around the reproductive organs after a dorsal incision in the abdomen in normal saline (in mM): 140 NaCl, 10 KCl, 1.6 CaCl_2_, 2 MgCl_2_, 44 D-glucose, and 2-[4-(2-hydroxyethyl)-1-piperazinyl] ethanesulfonic acid (HEPES); pH was adjusted to 7.4 with 2-amino-2-hydroxymethyl-1,3-propanediol (Tris(hydroxymethyl)aminomethane) (Tris). The left and right lateral oviducts connected to the common oviduct from the vitellarium were excised. Cell dissociation was performed enzymatically using the protease dispersion method described previously (15). The isolated single lateral oviduct myocytes were maintained in fresh saline at room temperature (23-27°C) and used within 12 h.

### 2.3 Electrophysiology

**Figure 1:**
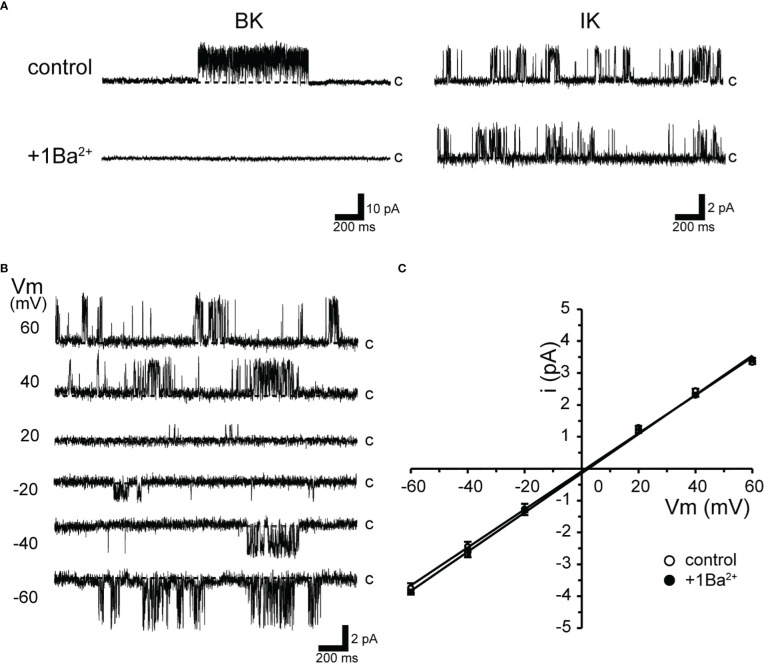
Ba^2+^-insensitive intermediate conductance single-K^+^ channel currents in isolated cricket oviduct cells. **(A)** Two types of single K^+^ channel current recordings from cell-attached patches under extracellular K^+^ concentration of 140 mM are shown. Representative single-channel current traces of large conductance Ca^2+^-activated potassium (BK) channels (left column) and intermediate conductance Ca^2+^-activated K^+^ channel (IK) channels are shown when held at ^+^40 mV. Notes at the BK channel currents are sensitive to extracellular (inside the pipette) 1 mM Ba^2+^ (1Ba). c indicates closed level. **(B)** Representative single-channel current traces at various membrane potentials (Vm) are shown in the figure. Current activity does not exhibit voltage sensitivity. c indicates closed level. **(C)** Average single-channel current (i)-Vm relationship (n = 6–12). Data points for Vm from −60 mV to ^+^60 mV were fitted by linear regression, yielding slope conductance values of 59.7 and 61.5 pS for the control and 1Ba, respectively. Cells isolated from a total of 153 animals were used in the experiment and 259 tests were performed for data collection.

**Figure 2:**
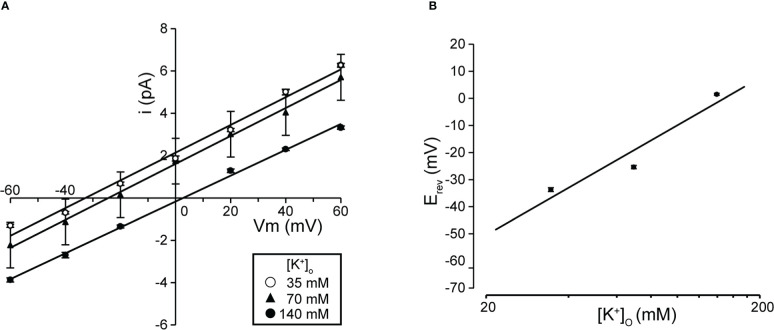
Single K^+^ channel I-V relationship at various extracellular K^+^ concentrations. **(A)** Mean linear current-voltage (I-V) data at various extracellular K^+^ concentrations ([K_+_]_o_ in mM): ●, 140; ▲, 70; ○, 35, from cell-attached patches. **(B)** Data show a semi-logarithmic plot of [K^+^]o against the difference between the reversal potential (⊿E_rev_) obtained at 140 mM and each K^+^ concentration. Reversal potential (E_rev_) was obtained by fitting the i-Vm relationship for each K^+^ concentration in A by linear regression. The slope of the regression line was 58.37 mV/decade. Cells isolated from a total of 53 animals were used in the experiment and 121 tests were performed for data collection.

**Figure 3:**
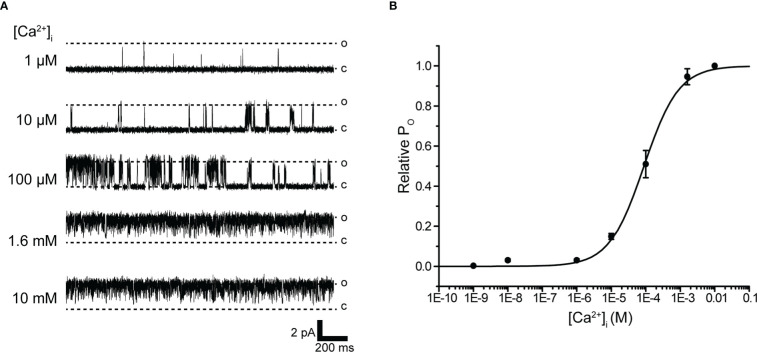
Intracellular Ca^2+^ concentration dependence of K^+^ channel activation. **(A)** Representative traces of single K^+^ channel currents recorded under various intracellular Ca^2+^ concentration ([Ca^2+^]_i_) conditions at Vm = 60 mV from excised inside-out patches. c and o indicate closed and open levels, respectively. **(B)** Average mean open probability (NP_O_)-[Ca^2+^]_i_ relationship of steady-state single-channel current. The data fit the Hill equation with EC_50_ of 88.5 ± 7.8 μM and Hill coefficient of 0.9. Cells isolated from a total of 14 animals were used in the experiment and 41 tests were performed for data collection.

**Figure 4:**
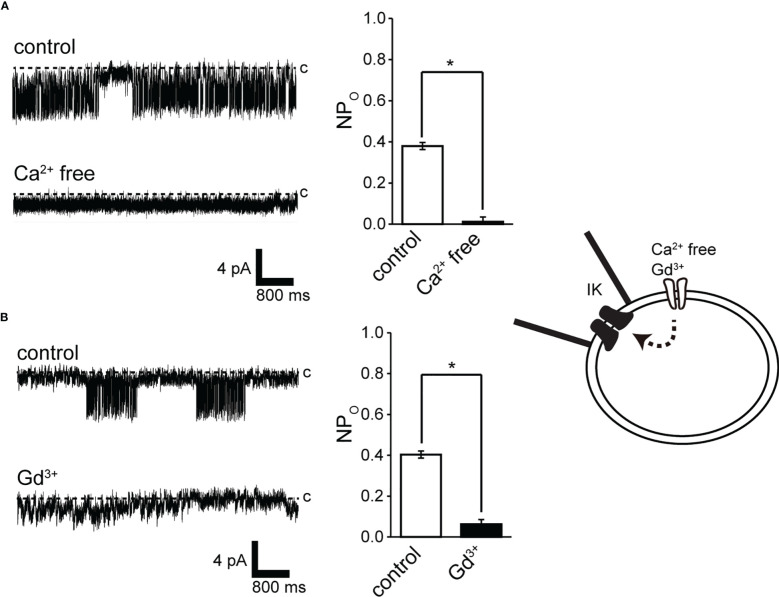
Effect of Ca^2+^ removal from the bath solution and Gd^3+^ application to the bath solution on IK channel activity. **(A, B)** (left) Representative IKchannel currents before (control) and after Ca^2^+ removal from the bath solution (Ca^2+^-free) or after 30 mM Gd^3+^ application to the bathsolution. The current was recorded from cell-attached patches at −140 mV. **(A, B)** (right) Average NP_O_ of IK channel current. *Significantly different (*P* < 0.05) from control values. Cells isolated from a total of 15 animals were used in the experiment and 25 tests were performed fordata collection.

**Figure 5:**
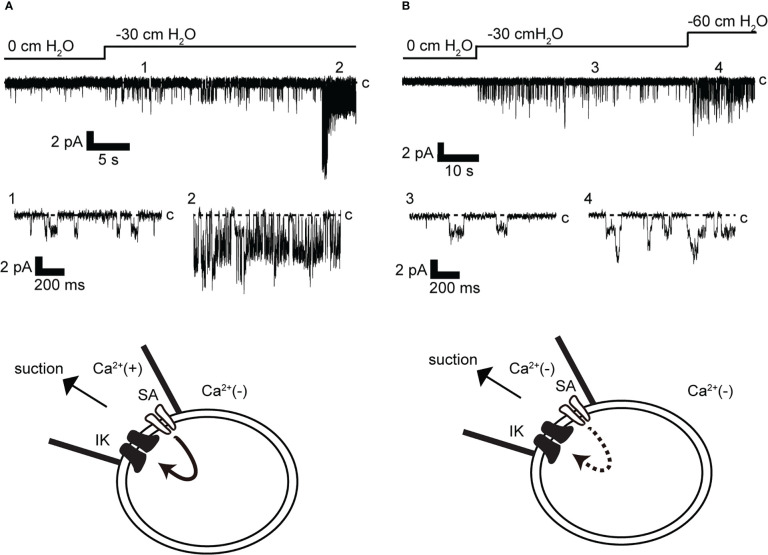
Effect of membrane stretch on IK channel activity. **(A, B)** Representative stretch-activated (SA) and IK channel currents were recorded at −140 mV from cell-attached mode (n = 12). Recordings were obtained after changing to a Ca^2+^-free bath solution. The pipette contains Ca^2+^ in A [Ca^2+^(+)] but does not contain Ca^2+^ in B [Ca^2+^(−)]. c indicates closed level. 1–4 show enlargements in lower and upper traces. Cells isolated from a total of 23 animals were used in the experiment and 43 tests were performed for data collection.

The cells were dropped on a glass-bottom dish containing the experimental solution, and the adhered cells were used for measurements. Cells were observed under an inverted microscope (IX70: Olympus, Tokyo, Japan). Currents from cells were amplified using Axopatch 200B (Axon Instruments/Molecular Devices, Union. City, CA, USA) and acquired using the Digidata1440 A/D converter (Axon Instruments/Molecular Devices). Experiments were performed at room temperature (22–27°C) using patch-clamp techniques for the cell-attached and excised inside-out mode. Patch electrodes were prepared from capillary tubes (Hemato-clad capillary; Drummond Scientific Co., Broomall, PA, USA) with a two-stage pipette puller (PC-10 Narishige, Tokyo, Japan) with a tip resistance of approximately 5–8 MΩ when filled with a solution for single-channel recordings. Current signals were filtered at 5 kHz with a four-pole Bessel filter and digitized at 10 or 20 kHz. pCLAMP (version 6, 7, 10, or 11; Axon Instruments/Molecular Devices) software was used for command pulse control, data acquisition, and analysis. A square voltage pulse of 3 mV, 10 ms, 10 Hz was applied as a ‘test pulse’ before the current amplitude measurement to ensure the accuracy of the measurement. A cell membrane resistance >1 gigaohm after application of the test pulse was considered a trial. Trials in which the current amplitude was observed within 10 mins after forming a stable gigaohm-seal were used for further analysis. The amplitude of single-channel currents, number of open channels, and mean open probability (NP_O_) were determined by a cursor on Clampfit, Fetchan, pStat, or using the single-channel search mode of the pCLAMP software. Data were also analyzed using the Origin (OriginLab Corp., Northampton, MA, USA), Excel (Microsoft, Redmont, WA, USA), and Sigma Plot (Systat Software, San Jose, CA, USA) software packages. For single-channel recordings to investigate the biophysical properties of ion channels (**Figures 1**, **2**), the external solution used contained a high potassium solution [(in mM) 140 KCl, 10 NaCl, 1.6 CaCl_2_, 2 MgCl_2_, 2 HEPES] that maintained the resting membrane potential at zero. The composition of the pipette solution was the same as that of the high-potassium bath solution with or without 1 mM Ba^2+^ used in experiments illustrated in **Figures 1**-**4A**, but Ca^2+^ was omitted for the experiments in **Figures 4B**, **5**. To test the K^+^ selectivity of isolated myocytes, 100, 70, and 35 mM KCl solutions were prepared by replacing KCl in the pipette solution with an equal amount of NaCl (11). To investigate the Ca^2+^ dependence of IK channels, Ca^2+^-free solution was obtained by omitting CaCl_2_ from the normal bath solution saline and adding 1 mM ethylene glycol-bis(2-aminoethylether)-N,N,N′,N′-tetraacetic acid (EGTA). The intracellular solution was balanced with 5 mM EGTA to maintain free Ca^2+^ concentrations between 1 nM and 10 μM. A bath solution with Ca^2+^ concentration of ≥100 μM was added by adjusting the amount of CaCl_2_ in normal saline. Extracellular effect of Ba^2+^ was tested by application in the pipette solution using the standard backfill method established previously (48). In brief, the tips of the electrodes were first filled with the normal pipette solution, then backfilled with the same solution containing a concentration of its inhibitors; a wait period of at least 10 min was ensured before data recording. Gd^3+^ was dissolved in water to prepare stock solutions, and aliquots were added to the perfusate. All inhibitor reagents used in the experiment were purchased from Sigma-Aldrich Corp. (St. Louis, MO, USA). All other reagents were purchased from Wako Pure Chemical Industries, Ltd. (Osaka, Japan).

### 2.4 Statistical analyses

All data are expressed as means ± standard error of mean (SEM). We accumulated data for each condition from at least three independent experiments. The numbers of animals, and trials used in each experiment are described in the figure legend. Statistical significance was evaluated by Student’s t-test for comparisons between two mean values to assess statistical significance using Excel (Microsoft Corp., Redmond, WA, USA) or Origin 8 (OriginLab Corp.) software. The data used for statistical analysis passed the Shapiro-Wilk normality test and the Levene equal variance test. For other correlation analyses, least-squares linear regression was performed using Excel (Microsoft Corp.). A *P*-value of <0.05 was considered significant.

## 3 Results

### 3.1 Single-channel recording of IK channels in isolated muscle cells

The top trace in **Figure 1A** shows single-channel activity with large and medium conductance in the steady state of oviduct myocytes. Single-channel currents in the left column in K^+^-rich recording conditions are consistent with our previous observations (11).

As shown in the upper traces of **Figure 1A**, both large (BK) and intermediate-sized single-K^+^ channel currents (IK) were observed either singly or multiples by trial when the membrane was held at +40 mV. During the course of this experiment, however, we newly found that the activity of this BK channel disappeared when 1 mM Ba^2+^ was added extracellularly. Thus, the extracellular addition of Ba^2+^ enabled selective recordings of the IK channels (**Figure 1A**, right column). Therefore, this study performed recordings using Ba^2+^ in a pipette to eliminate BK channel activity.

As shown in **Figure 1B**, when the membrane potential was held at depolarizing or hyperpolarizing potentials in cell-attached mode, the single-channel properties showed burst-like kinetics. The channel activity did not show voltage dependence (NP_O_ = 0.44 ± 0.03 and 0.51 ± 0.05 at +60 and −60 mV, respectively; n = 18). Single-channel currents recorded at membrane potentials from −60 mV to +60 mV showed a linear current-voltage (I-V) curve with a slope conductance of 61.5 pS (n = 12) (**Figure 1C**). In addition, even in the absence of extracellular Ba^2+^, IK currents recorded in patch membranes not coexisting with BK did not affect the slope conductance values (59.7 pS, n = 6).

We investigated the effects on single-channel conductance and reversal potential (E_rev_) observed in **Figure 1** by varying the extracellular K^+^ concentration. I-V relationships constructed from single-channel recordings at membrane potentials from −60 mV to +60 mV (**Figure 2A**) showed linear I-V relationships under three extracellular K^+^ concentrations. Linear analysis of the I-V relationship by least squares showed E_rev_s of −32.8, −24.5, and +2.4 mV for channel currents recorded under 35-, 70-, and 140-mM conditions, respectively. The slope of the E_rev_ change plotted against the change in extracellular K^+^ concentration was 58.4 mV per decade change in K^+^ concentration (**Figure 2B**). We next investigated the ion selectivity of the single-channel and recorded the effect on the E_rev_ by changing the extracellular K^+^ concentration. I-V relationships expressed from single-channel recordings at membrane potentials from −60 mV to +60 mV showed linear I-V relationships under three extracellular K^+^ concentrations. Linear analysis of the I-V relationship by least squares showed E_rev_s of −32.8, −24.5, and +2.4 mV for channel currents recorded under 35-, 70-, and 140-mM conditions, respectively (**Figure 2A**). The slope of the E_rev_ change plotted against the change in extracellular K^+^ concentration was 58.4 mV per decade change in K^+^ concentration (**Figure 2B**).

IK channels have the unique property of being activated by increases in [Ca^2+^]_i_ (28, 49). To directly evaluate the [Ca^2+^]_i_ dependence of K^+^ channels, we measured single-channel currents from excised inside-out patches of membranes to different concentrations of Ca^2+^-containing bath solutions. Single-channel currents recorded at various concentrations of Ca^2+^ bath solutions at +60 mV were enhanced with increasing [Ca^2+^]_i_ (**Figure 3A**). The relationship between relative Po and [Ca^2+^]_i_ was then fitted to the Hill equation, yielding a k-value of 88.5 μM and Hill coefficient of 0.9 (**Figure 3B**).

### 3.2 Functional coupling between IK and Ca^2+^ sources

Activation of Ca^2+^-dependent IK channels in excitable cells is critical for feedback control of both Ca^2+^ influx and cell excitability (27). To investigate the effect of Ca^2+^ on IK channel activity, we examined the effect of extracellular Ca^2+^ removal on IK channel activity.

Extracellular Ca^2+^ abolition suppressed IK channel activity after consistent IK channel activity (**Figure 4A**). We next investigated the pathways of extracellular Ca^2+^ influx and their effects on IK channel activity. Membrane stretching-induced extracellular Ca^2+^ influx pathways play a central role in myogenic muscle contraction (50). Gd^3+^-sensitive- and Ca^2+^ permeable-nonselective cation channels activated by membrane stretching (SA channel) during muscle contraction are functionally expressed in cricket oviduct cells (15). In support of these evidences, Gd^3+^ administration suppressed IK channel activity (**Figure 4B**). We further investigated the proximity of IK and SA channels within microdomains by observing the effect of membrane stretch on IK channel activity.

To eliminate the possibility of Ca^2+^ influx from the bath solution, the bath solution was replaced with a Ca^2+^-free solution before the recording (**Figure 5**, lower panel). Recordings were obtained with and without Ca^2+^ in the pipette. This restricted Ca^2+^ access to the patch membrane only from the pipette solution. In addition, the recording membrane voltage was maintained at −140 mV, allowing simultaneous measurement of low-conductance SA channel currents (~13 pS) (15) and IK channel activity. As shown in **Figure 5A**, when Ca^2+^ was contained in the pipette, SA channel activity increased in response to membrane stretching caused by mechanical stimulation in response to a negative pressure of −30 cm H_2_O in the pipette (**Figure 5A**). This mechano-stimulated single-channel activity is consistent with results in our previous report (15). Around 30 s after SA activity was recorded, IK channel activity was observed. It should be noted that SA and IK channels can be observed individually due to their different conductance and mean opening time. The conductance and mean opening time of the SA channel were 14 pS and 5.4 ms (n = 40) and those of IK channel were 61.5 pS and 1.2 ms (n = 48), respectively. The same SA channel activity as in **Figure 5A** was observed with −30 cm H_2_O suction wherein Ca^2+^ was not contained in the pipette (**Figure 5B**). Under this condition, even if the suction was further increased to −60 cm H_2_O, SA channel activity increased, but IK channel activity was not observed (n = 12).

**Figure 6:**
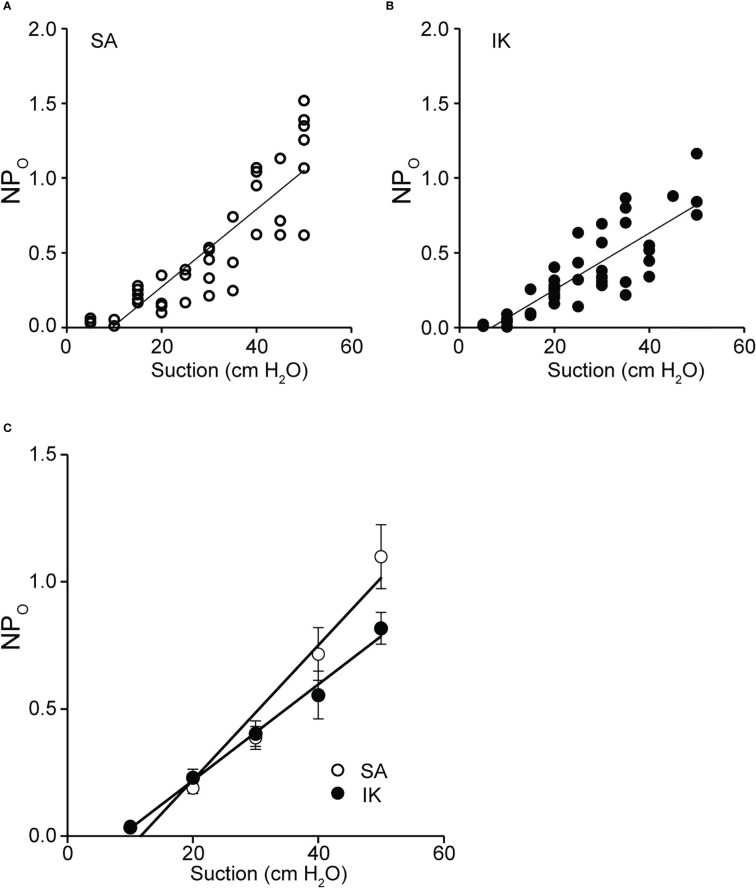
Activity relationship between SA and IK due to membrane stretch. **(A)** Scatter plot of NP_O_ (SA channel activity) was recorded at −140 mV versus membrane suction (n = 48). **(B)** Scatter plot of NP_O_ (IK channel activity) recorded at −140 mV versus membrane suction (n = 40). **(A, B)** The values for each relationship were obtained by fitting them with linear regression. The slopes obtained from plots A and B revealed 0.026 and 0.019 cm H_2_O, respectively. Relationship between NP_O_ and suction, and analyses of the correlation function revealed values of 0.84 and 0.87 from the values of A and B, respectively, indicating a strong positive correlation. **(C)** Scatterplots of mean NP_O_ versus membrane suction (n = 7) for SA and IK, recorded in **A** and **B**, are shown. The correlation coefficient between SA and IK was 0.99. Cells isolated from a total of 83 animals were used in the experiment and 108 tests were performed for data collection.

We analyzed the effect of membrane stretching on the activity of SA and IK channels, as observed in **Figure 5A**. As shown in **Figures 6A**, **B** channel activity increased with the strength of membrane stretching, and positive correlation coefficients of 0.987 and 0.996 were calculated for SA and IK channels, respectively. Furthermore, the average value at 10 cm of suction strength was analyzed. It was revealed that SA and IK channels increased NPo by 0.027 and 0.019, respectively, with respect to membrane stretching strength of 1 cm H_2_O (**Figure 6C**).

## 4 Discussion

In this study, we performed patch-clamp electrophysiology to characterize the functional expression of IK channels in cricket oviduct cells. We demonstrated for the first time that IK channels are endogenously expressed in this cell type. A series of single-channel activity recordings showed these channels as IK channels according to classification criteria such as K^+^ selectivity, conductance, voltage independence, and intracellular Ca^2+^ sensitivity (**Figures 1**–**3**). These cumulative properties are closely consistent with the intermediate conductance of IK channels observed in vertebrates, including humans and mice (22, 24, 51). Concerning Ca^2+^ sources that activate IK channels, extracellular Ca^2+^ influx was essential for IK channel activity (**Figure 4A**). Furthermore, we observed that Ca^2+^ influx from Gd^3+^-sensitive SA channels affected IK channel activity (**Figures 4**, **5**). These observations imply that SA channel-mediated Ca^2+^ influx induced by the membrane mechanical stretch, may trigger IK activity, which leads to an increase in the amount of Ca^2+^ influx through SA channels *via* an increase in the driving force for Ca^2+^ through membrane hyperpolarization. Indeed, in our previous muscle strength measurement study, hyperpolarization induced by IK channels may produce a voltage-independent driving force for Ca^2+^ influx in SA channels (9, 47). Furthermore, simultaneous single-channel current measurement of SA and IK channels in response to membrane stretching stimulation provided evidence for the proximity of IK and SA channels in patch membrane microregions (**Figures 5**, **6**).

K_Ca_ is classified into three types according to the magnitude of conductance. Intermediate conductance calcium-activated potassium (IK) channels have a range of 20–85 pS between BK and SK channels (22–24). BK channels have activity defined by both voltage and intracellular Ca^2+^, whereas small and moderate K_Ca_ channels are sensitive to changes in intracellular Ca^2+^ and are activated in a voltage-independent manner.

Gating of IK channels is achieved by submicromolar changes in cytosolic Ca^2+^ levels (K_D_ = 0.5 µM) (52). The intracellular Ca^2+^ sensitivity of IK channels in cricket muscle cells showed a median effective concentration (EC_50_) of 88.5 µM (**Figure 3**). This value can be classified as sensitive to intracellular Ca^2+^ in terms of vertebrate reports (52). Based on structural information, the current assumption on the mechanism of IK channel activation suggests that gating is influenced by calmodulin (CaM) when [Ca^2+^]_i_ increases (53, 54). The importance of CaM is also supported by studies on IK activity shifts that impair function by CaM EF hand mutations (55). Therefore, the low intracellular Ca^2+^ sensitivity of IK channels presented in this study may be due to the loss of CaM performed in the inside-out mode.

Activation of IK channels reflects the associated Ca^2^-permeable ion channel activation threshold. For example, the Ca^2+^ influx pathway that activates IK is SA (56), Orai (57), SOC (store-operated calcium) (58), CRAC (calcium release-activated calcium channel) (59), TRP (transient receptor potential) C1 (60), TRPV4 (61, 62), TRPV6 (29), TRPM7 (63), and TRPA1 (64) channel show activity with hyperpolarization.

This study performed simultaneous single current measurements of SA and IK channels with a small tip within sub-μm of a patch pipette in cell connection mode, directly demonstrating their proximity (**Figures 5**, **6**). From these observations, IK channels were sufficiently characterized to construct microdomains in this study (27). It should be noted, however, that a time lag of ~30 s was observed between SA channel activity and increased IK channel activity. This delay implies that the global Ca^2+^ rise in cells required for IK channel activity involves Ca^2+^ release from intracellular stores.

For K_Ca_ and voltage-gated calcium channel (Ca_V_), the concept of 1:1 stoichiometry evaluates proximity due to physical interactions. However, it has been suggested that the stoichiometry of physical interaction and functional coupling is not congruent (27). **Figure 6** demonstrates that the activation ratio of SA : IK channel was 3:2 when the strength of mechanical membrane stretching exceeded 20 cm H_2_O. The results are the first to demonstrate functional measurements with two single-channel activities and provide quantitative activity ratios in function, providing insights into computational science.

The stretch-sensitive, non-selective cation channel in this study can be activated around 20 cm H_2_O and affects IK channel activity by supplying intracellular Ca^2+^ (**Figure 6**) (15). In the esophagus and uterus of vertebrates, membranes are stretched due to increased blood pressure and the passage of eggs. It has been reported that the mechanical force measured using a manometry catheter is ~20 cm H_2_O under normal conditions (20, 65, 66). Mechanical forces above 20 mmHg are applied during peristalsis and solids outflow. According to the results of this study, IK channel activity increased when a membrane tension of 20 cm H_2_O (14.7 mmHg) was applied (**Figure 5A**). In invertebrates, intraluminal pressure has not been measured. However, physical stretching into the lumen as it passes through the oviduct suggests that the influx of Ca^2+^ causes muscle contraction, leading to initial stretching. Activation of the IK channel increases Ca^2+^ influx through the Ca^2+^ channel by increasing the driving force of the membrane potential, which may be a sufficient condition to trigger myogenic peristalsis as the egg passes. This mechanism has also been suggested for Ca^2+^ influx and IK channel activity occurring during crop filling and ejection processes in hypercontractile crop muscles in the blowfly *Phormia regina* (Meigen) (67).

Activation of IK channel is associated with more extended-lasting activations, such as AVD (apoptotic volume decreases) (68), RVD (regulatory volume decreases) (56), and cell migration (69). BK channels, in combination with Ca_V_s (voltage-gated calcium channels) and NMDARs (N-methyl-D-aspartate receptors), play a fundamental role in the short-term regulation of depolarizing nervous system firing (27), thus, contrasting with IK channels. Therefore, Ca^2+^ sources and CaK (Ca^2+^-activated K^+^) channels may function in unique combinations. When hypotonic mechanical stimulation is applied to the cricket oviduct, binding functions of the SA and IK channels are related, and it is possible that they are involved in muscle contraction that takes seconds with hyperpolarization (9, 47).

In conclusion, the characterization of single IK channels in oviduct myocytes enabled us to investigate their functional relevance to Ca^2+^ sources. Furthermore, IK channels functionally coupled with rhythmic contraction-producing SA channels form a driving force for increased Ca^2+^ influx with periodic hyperpolarization. We propose that cricket muscle cells are involved in spontaneous contraction *via* the IK and SA channel microdomains.

## Data availability statement

The original contributions presented in the study are included in the article/supplementary materials. Further inquiries can be directed to the corresponding authors.

## Author contributions

TN and MY: conceptualization and design of study. TN and KS-N: performing experiments and analyses, writing, and editing. TN: writing original draft. MY: supervision. TN: funding acquisition. All authors contributed to the article and approved the submitted version.
